# Evaluation of urinary podocin and nephrin as markers of podocyturia in dogs with leishmaniosis

**DOI:** 10.1186/s13071-024-06510-3

**Published:** 2024-10-08

**Authors:** Valeria Pantaleo, Tommaso Furlanello, Erika Carli, Laura Ventura, Laia Solano-Gallego

**Affiliations:** 1 San Marco Veterinary Clinic and Laboratory, Veggiano, Padua, Italy; 2 Department of Statistical Sciences, University of Padova, Padua, Italy; 3 Departament de Medicina i Cirurgia Animals, Universitat Autònoma de Barcelona, Bellaterra, Barcelona, Spain

**Keywords:** Canine, Glomerular disease, *Leishmania infantum*, Renal markers

## Abstract

**Background:**

Renal disease is the main cause of death in canine leishmaniosis. Detection of an active glomerular injury is important to identify early renal damage and to prevent the development of chronic kidney disease. Podocyturia can indicate renal injury, and podocyte-associated molecules such as podocin and nephrin can be used to identify podocyturia. The purpose of the study was to evaluate urinary podocin and nephrin concentrations in dogs with leishmaniosis as markers of podocyturia.

**Methods:**

A total of 35 healthy dogs and 37 dogs with leishmaniosis were enrolled in the study. Dogs with leishmaniosis were classified according to the staging of the International Renal Interest Society (IRIS). Urinary podocin and nephrin concentrations were measured in all dogs with a validated enzyme-linked immunosorbent assay test and normalized to creatinine (uPoC and uNeC, respectively). The demographic, clinical, and laboratory data from both groups were analyzed and compared. Subsequently, the laboratory results were analyzed and compared according to IRIS staging in dogs in IRIS stage I and dogs in IRIS stage II + III + IV. The Pearson’s correlation test evaluated the relationship between urinary markers of podocyturia.

**Results:**

Compared with healthy dogs, lower urinary podocin [median values (IQR): 15.10 (11.75–17.87) ng/ml versus 8.63 (7.08–13.56) ng/ml; *P* < 0.01] and nephrin [median values (IQR): 3.2 (3.62–5.43) ng/ml versus 2.67 (2.06–3.44) ng/ml; *P* < 0.01] were found in infected sick dogs. No significant differences were observed in the uPoC and uNeC between the two groups. Urinary nephrin and podocin concentrations were higher in healthy dogs and in dogs in IRIS stage I (both *P* < 0.05) compared with dogs in IRIS stages II + III + IV. No significant differences were found for uPoC and uNeC between healthy dogs and dogs with leishmaniosis in different IRIS clinical stages.

**Conclusions:**

Dogs with leishmaniosis had a low concentration of podocin and nephrin in more advanced IRIS clinical stages, when kidney disease was more severe compared with healthy dogs and dogs in IRIS stage I with mild disease. Urinary nephrin was detectable for the first time in healthy non-infected dogs.

**Graphical Abstract:**

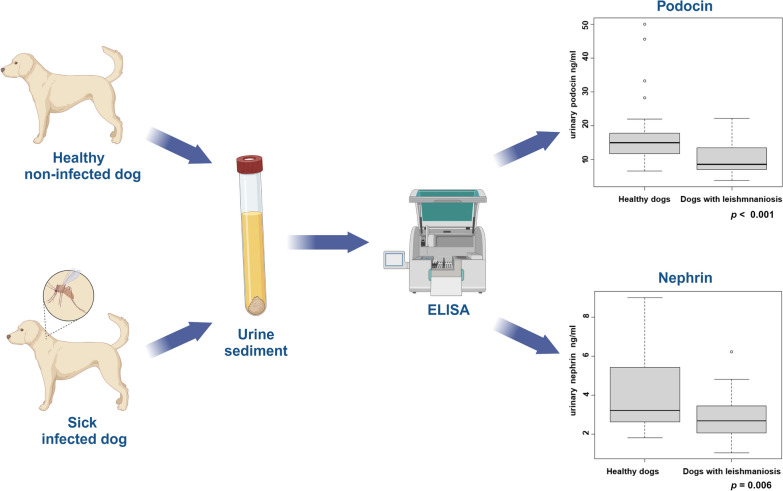

**Supplementary Information:**

The online version contains supplementary material available at 10.1186/s13071-024-06510-3.

## Background

Canine leishmaniosis (CanL) is a parasitic disease caused by the protozoan *Leishmania infantum* (*L. infantum*). Infection in dogs may be subclinical or presented as a self-limiting disease, or as a severe and sometimes fatal disease [1, 2]. A LeishVet staging system has been proposed to define the severity of the disease and facilitate appropriate treatment and patient monitoring [3]. Because severe disease can be associated with renal dysfunction of various degrees, if kidney disease is not diagnosed in the early stages, it can progress to chronic renal failure, which is considered the main cause of mortality in CanL [4, 5].

Despite the high prevalence of renal disease in infected dogs [6, 7], laboratory findings of renal disease are typically variable, with different degrees of azotemia and/or proteinuria [5, 8, 9]. In CanL, renal disease is primarily of glomerular origin, involving different forms of glomerulonephritis [6, 7]. Initially, mild proteinuria appears, over time it worsens, and with disease progression secondary tubulointerstitial lesions and azotemia develop [9, 10]. Because the severity of kidney disease reduces treatment options and survival [4, 11, 12], the identification of new markers of early renal damage could lead to a more favorable prognosis [13–18]. The activity of some urinary markers to detect glomerular damage, such as the urinary immunoglobulin-G-to-creatinine ratio, the urinary C-reactive-protein-to-creatinine ratio and the urinary ferritin-to-creatinine ratio, has been investigated in non-azotemic and non-proteinuric dogs with leishmaniosis treated with meglumine antimoniate and allopurinol, with promising results [19]. More research is needed before these glomerular markers can be routinely recommended for early recognition of glomerular damage, consequently, proteinuria remains the first clinicopathological finding of glomerulopathy during CanL [10, 20].

In this context, the detection of early glomerular injury is of primary importance and the presence of podocytes in urine is a potential tool to diagnose glomerular damage at the beginning of the disease [21]. Podocytes are highly specialized glomerular epithelial cells involved in selective plasma filtration and the formation of primary urine. Podocyturia can occur naturally in humans [22], dogs [23, 24], and horses [25], and various pathological processes can cause podocytes tearing and excretion in the urine with an increased extent of podocyturia [26]. Since podocytes do not regenerate, their loss is irreversible [27]. In human beings, 20–40% loss of glomerular podocytes has been shown to lead to glomerulosclerosis [28, 29], with proteinuria as one of the main consequences [30]. The presence of podocytes in urine can be determined by the detection of podocyte-associated molecules, such as podocin, nephrin, podocalyxin, and synaptopodin. Podocyturia was previously assessed by urinary podocin concentration using an enzyme-linked immunosorbent assay (ELISA) test in dogs with chronic kidney disease and degenerative mitral valve disease, with greater podocyturia in these two groups compared with healthy dogs [23]. The expression of the nephrin gene has been evaluated in dogs with chronic kidney disease associated with leishmaniosis in urinary sediments, with dogs in advanced stages of kidney disease having lower expression of nephrin than dogs in the initial stages [31]. A recent study evaluated the presence of nephrin and podocin mRNA in urinary sediment in dogs with chronic kidney disease not relative to *L. infantum* infection and healthy dogs [24]. According to the stage of the disease established by International Renal Interest Society (IRIS) [32], an increase in podocyturia in early stages and a reduction in advanced stages were observed [24]. In human medicine, urinary nephrin concentration has been evaluated under different clinical conditions to detect podocyturia, and the most widely used method for its measurement was various commercially available ELISA kits [33–37]. To our knowledge, urinary podocin and nephrin have never been measured with an ELISA test in CanL and human leishmaniasis despite their potential accessibility and cost-effectiveness. The aims of the present study were: (1) to evaluate and compare the concentrations of urinary podocin and nephrin as markers of podocyturia in healthy dogs and in dogs with leishmaniosis according to IRIS clinical staging with a commercial ELISA test and (2) to evaluate the correlation between urinary podocin and nephrin concentration and urinary podocin-to-creatine ratio and urinary nephrin-to-creatinine ratio and some renal and urinary markers in healthy dogs and in dogs with leishmaniosis according to IRIS clinical staging.

## Methods

### Dogs

This is a cross-sectional study that includes clinical and laboratory data belonging to 72 client-owned dogs that were admitted due to various medical reasons to the San Marco Veterinary Clinic (Veggiano, Italy) between November 2022 and January 2023.

The dogs were divided into two groups: (1) dogs with leishmaniosis (*n* = 37) and (2) healthy dogs (*n* = 35). The dogs were diagnosed with clinical leishmaniosis on the basis of compatible clinical signs, clinical pathological findings, a positive *L. infantum* ELISA serology, and a positive *Leishmania* real-time polymerase chain reaction (q-PCR) in the bone marrow [3, 4]. To be enrolled in the study, the following inclusion criteria were required for dogs with leishmaniosis: (1) no current anti-*Leishmania* treatment; (2) availability of exams including complete blood count (CBC), serum biochemistry, coagulation profile, and urinalysis; (3) absence of *Dirofilaria immitis* antigen (Filarcheck 96, biopronix by Agrolabo, Italy), absence of *Anaplasma phagocytophilum, Ehrlichia canis*, and *Rickettsia conorii* antibodies (semiquantitative immunofluorescence by MegaFLUO ANAPLASMA ph. MEGACOR; MegaFLUO EHRLICHIA canis MEGACOR; MegaFLUO RICKETTSIA conorii MEGACOR; Hörbranz, Austria); (4) inactive urine sediment; (5) no other concurrent diseases; and (6) no administration of any type of drug in the previous 3 months. The inclusion criteria for healthy dogs were: (1) routine check as the reason for the visit to the clinic, (2) absence of any clinical signs of illness on physical examination, (3) normal results in all laboratory tests including CBC, serum biochemistry, coagulation profile, and urinalysis, and (4) a negative *L. infantum* ELISA serology. Of the 35 healthy dogs included in the study, 20 dogs as annual recheck were tested at the time of their evaluation for *D. immitis* antigen and *A. phagocytophilum, E. canis*, and *R.  conorii* antibodies as described above and resulted negative. The remaining 15 dogs were tested 6 months before and resulted all negative at that time.

An abdominal ultrasound was performed in all dogs with leishmaniosis with the ACUSON Juniper 2.0 (Siemens Medical Solution, USA) using a 7.3 MHz microconvex and linear probe. The following ultrasonographic findings such as increased renal cortical and/or medullary echogenicity, decreased renal corticomedullary distinction, irregular renal margins, and small-sized kidneys were recorded and considered compatible with chronic kidney disease [38, 39].

At the time of diagnosis, all dogs with leishmaniosis were classified according to the IRIS recommendations for chronic kidney disease [32].

Previous history, physical exam, creatinine, symmetric-dimethylarginine (SDMA), urine-specific gravity (USG), and urine protein-to-creatinine ratio (UPC) on a fasted state were available for all dogs with leishmaniosis before the admission to the hospital. Of the 37 dogs with leishmaniosis included in the study, previous history, physical exam, creatinine, SDMA, USG, and UPC on a fasted state were available for 24 dogs 3 and 6 months before admission to the hospital, for 1 dog 7 months before admission to the hospital, for 7 dogs 2 weeks before admission to the hospital, and for 5 dogs 3 days before admission to the hospital.

As part of the physical examination, after a 20-min adaptation period to the environment, systolic blood pressure (SBP) was measured in each dog with the automated blood pressure monitor for companion animals SunTech Vet 20 (SunTech Medical Inc., USA). After discarding the first measurement, the average value of four consecutive measurements was recorded. The following four measurements were similar to each other (with a maximum difference of 2–3 mmHg between the various measurements). All exams were performed in the morning after 12 h of fasting without pharmacological or other restraint.

### Blood tests

All clinicopathological tests were performed at the San Marco Laboratory (Veggiano, Italy). A blood sample was collected by cephalic, saphenous, or jugular venipuncture in a 10 ml sterile plastic syringe, and 2 ml of blood was transferred to plastic tubes containing K_3_-EDTA for a CBC performed on an automated hematology analyser (ADVIA 2120i, Siemens, Germany) with a blood smear microscopic evaluation. Then 4 ml of blood was placed in serum glass tubes for chemistry analysis performed in an automated biochemical analyzer (Atellica^®^ Solution, Siemens, Germany), and the following parameters were evaluated: white blood cell concentration (WBC), paraoxonase-1 (PON-1), haptoglobin (Hp), ferritin (Ft), C-reactive protein (CRP), total iron-binding capacity (TIBC), iron, albumin (Alb), globulins (Glob), glucose (Gluc), gamma-glutamyl transferase (GGT), amylase, lipase, sodium (Na), urea, and creatinine (Cr). In addition, symmetric-dimethylarginine (SDMA) was measured with a canine SDMA ELISA test (Eurolyser Diagnostica GmbH, Salzburg, Austria).

To detect *L. infantum* antibodies, a *Leishmania* ELISA test was performed according to the manufacturer’s instructions (VetLine *Leishmania*, *Leishmania* ELISA test, NovaTec Immunodiagnostica GmbH, Dietzenbach, Germany). The result of the *Leishmania* ELISA test was considered negative if the antibody level was < 9%, doubtful if the antibody level was 9–11%, and positive if the antibody level was > 11%.

### Urine test

Urine was collected at the time of the visit (in the morning, after blood sampling) by free catch in a sterile container in all dogs. A total volume of 10 ml of urine was obtained during spontaneous urination; 7 ml of urine was used for urinalysis and urinary chemistry performed on an automated urine analyzer (CLINITEK Novus^®^, Siemens, Germany) and on an automated biochemical analyzer (Atellica^®^ Solution, Siemens, Germany), respectively. Whole urine was used for urinalysis and urine-specific gravity (USG) measurement with test strips (CINITEK Novus Pro12 Urinalysis Cassette, Siemens, Germany), determination of urine protein-to-creatinine ratio (UPC) (calculated by dividing the concentration of urinary proteins by the concentration of urinary Cr concentration), and urinary chemistry. Urine proteins (UPs) were measured in an automated spectrophotometer (Atellica^®^ Solution, Siemens, Germany) using pyrogallol red [Atellica CH urinary/cerebrospinal fluid protein (UCFP), Siemens Healthcare Diagnostics Inc., USA] as previously described [40, 41], and uCr with a modified Jaffe method (Siemens Healthcare Diagnostics Inc., USA). Samples were automatically prediluted 1:5 to fit the linearity of the method according to manufacturer’s instructions. Urinary sediment was examined by a clinical pathologist with an optical microscope and only dogs with inactive urine sediment [< 5 white blood cells per high-power field (hpf), < 5 red blood cells/hpf or no visible bacteria] were considered for UPC, urinary podocin, and nephrin measurements. In addition, no sperm cells were observed in the urinary sediment of any healthy or leishmaniotic dog. The following parameters in the urine were evaluated: USG, UPC, fractional excretion of sodium (FeNa) calculated according to the equation as follows: FeNa = uNa × serum Cr/uCr × serum Na [42], urinary amylase-to-creatinine ratio (uAm/Cr), urinary ferritin-to-creatinine ratio (uFerr/Cr), urinary gamma-glutamyltransferase-to-creatinine ratio (uGGT/Cr), urinary glucose-to-creatinine ratio (uGlu/Cr), and urinary creatinine (uCr).

### Podocin and nephrin determinations

A total of 2 ml of urine was centrifuged at low speed (1500 *g* × 5 min) to prevent podocyte damage and 2 aliquots of 0.25 ml of urine sediment sample from each dog were stored at −80 °C until podocin and nephrin ELISA tests (Canine Podocin ELISA test and Canine Nephrin ELISA test, MyBioSource.com, San Diego, California, USA) were performed. Urinary podocin and nephrin were measured in urine sediment as previously reported [23, 43]. Once all urine samples were collected, ELISA tests were performed to detect urinary podocin and nephrin concentrations according to the manufacturer’s protocol. Briefly, the ELISA plate was set for blank, standard, and sample wells. The blank control well was assigned with 100 µl of phosphate buffered saline, the standard with 100 µl of standard product, and sample wells with 100 µl of sample test. An additional 10 µl of balance solution was distributed only in 100 µl samples and mixed well, 50 µl of conjugate reagent was added to each well except the blank well. At this point, the plate was covered and incubated for 60 min at 37 °C. Then, all wells were washed five times, treated with 50 µl of two substrate solutions (A and B), and the plate was kept in the dark and incubated for 15 min at 37 °C for the development of the color. At the end of the incubation, 50 µl of stopping solution was added to each well to end the reaction. The optical density was measured with a microplate reader at a wavelength of 450 nm and the standard curve was prepared, on the basis of which the podocin and nephrin concentrations in the samples were calculated. The standard curve for podocin concentration ranged from 2.5 to 50 ng/ml and for nephrin concentration ranged from 1.0 to 25 ng/ml and was calculated using a computer-generated four-parameter logistic curve-fit with program test result of the automated analyzer Stratego (Futurlab^®^, Limena, Padova, Italy). Each sample was measured in duplicate, and the average values obtained were expressed in ng/ml. Once the urinary podocin and nephrin concentrations were determined, their levels were assessed relative to the urinary creatinine concentration as the urinary podocin-to-creatinine ratio (uPoC) and the urinary nephrin-to-creatinine ratio (uNeC) because a quantitative urinary podocin and nephrin depend strongly on the degree of urine concentration [22, 44].

### Evaluation of *Leishmania* parasitic load

*Leishmania* q-PCR was measured in bone marrow, whole blood, and whole urine of all dogs with leishmaniosis. Bone marrow aspirates were obtained from the costochondral junctions using an 18-gauge needle connected to a 10-ml syringe according to the protocol described by Paparcone and colleagues for the diagnosis of CanL [45]. DNA extraction was performed using a High Pure PCR Template Preparation Kit (Roche Science Applied) and performed according to the manufacturer’s protocol. Real-time PCR was performed using LightCycler FastStart DNA Master^PLUS^ Hybridization Probes (Roche, Mannheim, Germany), employing a LightCycler version 3.5.17 instrument (Roche, Mannheim, Germany). Commercial *L. infantum* primers and hybridization probes LC set (TIB Molbiol, Genova, Italy) that amplified a fragment of the kinetoplast minicircle were used. Thermal cycling was carried out according to the manufacturer’s instructions (TIB Molbiol). Positive and negative controls were used in all q-PCR runs as previously reported [46]. To be considered positive, > 100 copies of kinetoplast/ml should be detected in bone marrow, whole blood and urine.

### Statistical analysis

Quantitative data were expressed as mean ± standard deviation, if normally distributed, and as median and interquartile range (IQR), if not normally distributed. Categorical variables were expressed as counts and percentages in each category. The normality assumption for quantitative variables was assessed with the Shapiro–Wilk test.

Comparisons of quantitative variables in healthy and infected dogs were analyzed with the two-sample *t*-test, the Welch’s *t*-test, or the Mann–Whitney rank sum according, respectively, to the assumptions of normality and homoscedasticity. The associations between qualitative variables and healthy and infected dogs were evaluated with the Pearson’s chi-squared test or the Fisher’s exact test.

Differences between quantitative variables, such as urinary podocin, urinary nephrin, uPoC, and uNeC, were evaluated in healthy and all the infected sick dogs and also referred to the IRIS staging groups using the non-parametric ANOVA Kruskall–Wallis test, using post hoc analyses on the basis of the Bonferroni correction. For this part of the analysis, infected sick dogs previously classified in IRIS stages II, III, and IV were aggregated to obtain a group sufficiently numerous to be compared.

The relationships between urinary podocin, urinary nephrin, uPoC, and uNeC and SBP and other urinalysis parameters were expressed using the Pearson’s correlation test to assess whether its concentration in the urine was similar, in all dogs and in healthy and leishmaniotic dogs.

To evaluate the role of urinary podocin and urinary nephrin as markers of renal damage during *Leishmania* infection, a logistic regression model was fitted through a backward variable selection. The goodness-of-fit was assessed with the area under the receiver-operating characteristic curve (ROC-AUC) and the Hosmer–Lemeshow test. The statistical significance was declared for *P*-value < 0.05. Statistical analysis was implemented using R (https://www.r-project.org/).

## Results

The demographic and clinical data and blood and urine analysis results of healthy dogs and dogs with leishmaniosis are summarized in Tables 1, 2, and 3. All data supporting the main conclusions are displayed in Additional file 1 (Dataset S1: signalment, clinical data, and serum and urinary parameters including podocin and nephrin).

**Table 1:** Demographic and clinical data of healthy dogs and dogs with leishmaniosis

Parameter (units)	Healthy dogs	Dogs with leishmaniosis	Statistical analysis
	*n* = 35	*n* = 37	
Female/male	18/17	22/15	*χ*^2^ = 0.20, *df* = 1, *P* = 0.65
Breed/ mixed breed	27/8	23/14	*χ*^2^ = 1.26, *df* = 1, *P* = 0.21
Neutered/intact	20/15	16/21	*χ*^2^ = 0.89, *df* = 1, *P* = 0.35
Age (months)	70.03 ± 35.22	72.49 ± 31.38	*t* = −0.31, *df* = 1, *P* = 0.75
Body weight (kg)	24.34 ± 13.16	22.52 ± 11.27	*t* = 0.63, *df* = 1, *P* = 0.53
BCS (1–9)	5 (5–5)	5 (5–5)	*U* = 773.5, *Z* = 1.87, *P* = 0.061
Heart rate (bpm)	100 (86–111)	120 (100–140)	*U* = 402.5, *Z* = −2.76, *P* = 0.005*
Respiration rate (rpm)	30 (24–34)	32 (28–40)	*U* = 460, *Z* = −2.11, *P* = 0.033*
Systolic blood pressure (mmHg)	142 (129–150)	150 (140–170)	*U* = 434, *Z* = −2.41, *P* = 0.016*
Bone marrow *Leishmania* q-PCR (k/ml) and frequency of positivity	–	8.5 × 10^7^ (2760–6.8 × 10^9^), 37/37 (100%)	*–*
Blood *Leishmania* q-PCR (k/ml) and frequency of positivity	–	5400 (0–1.5 × 10^7^), 29/37 (78%)	*–*
Urine *Leishmania* q-PCR (k/ml) and frequency of positivity	–	0 (0–2.1 × 10^6^), 9/37 (24%)	*–*

Data are expressed as counts, mean ± standard deviation, or median and interquartile range

*BCS* body condition score, *Kg* kilograms, *bpm* beats per minute, *rpm* breaths per minute, *mmHg* millimeters of mercury, *k/ml* kinetoplast copies per milliliters, *χ*^2^ chi-squared test, *df* degrees of freedom, *t* *t*-test, *U* Mann–Whitney *U*-test, *Z* Mann–Whitney *Z*-score

^*^Statistically significant differences between healthy dogs and dogs with leishmaniosis

**Table 2:** Blood analysis of healthy dogs and dogs with leishmaniosis

Parameter (units)	Healthy dogs	Dogs with leishmaniosis	Statistical analysis
(Reference interval)	*n* = 35	*n* = 37	
WBC (10^3^/µl)			
(6.52–10.56)	7.54 (6.45–9.41)	7.22 (6.07–10.2)	*U* = 690, *Z* = 0.47, *P* = 0.63
CRP (mg/dl)			
(0.01–0.07)	0.01 (0.01–0.14)	1.74 (0.38–4.91)	*U* = 163, Z = 5.46, *P* < 0.001*
PON-1 (IU/l)			
(3.02–4.71)	3.60 (3.4–4.18)	3.37 (2.94–3.88)	*U* = 833.5, *Z* = 2.09, *P* = 0.036*
Ft (ng/ml)			
(80–272)	231 (205–263)	714 (539–1176)	*U* = 111.5, *Z* = −6.04, *P* < 0.001*
Hp (mg/dl)			
(2–165)	21 (4–62.5)	198 (93–282)	*U* = 129.5, *Z* = −5.84, *P* < 0.001*
Iron (μg/dl)			
(95–213)	95 (126–172)	81 (61–119)	*U* = 1038, *Z* = 4.39, *P* < 0.001*
TIBC (μg/dl)			
(336–424)	363 (340.5–379.5)	300 (238–350)	*U* = 1006.5, *Z* = 4.04, *P* < 0.001*
Alb (g/dl)			
(2.9–3.5)	3.2 (3.05–3.35)	2.4 (2.1–2.9)	*U* = 1107.5, *Z* = 5.18, *P* < 0.001*
Glob (g/dl)			
(2.9–3.4)	3.24 ± 0.31	5.24 ± 1.75	*t* = −6.84, *df* = 38, *P* < 0.001*
Gluc (mg/dl)			
(85–120)	99 ± 6.83	101.2 ± 9.63	*t* = −1.05, *df* = 70, *P* = 0.30
GGT (IU/l)			
(1–4.9)	4 (3.45–4.55)	3.5 (2.4–4.3)	*U* = 835.5, *Z* = 2.11, *P* = 0.034*
Amylase (IU/l)			
(176–764)	579 (468.5–792)	1281 (1002–1612)	*U* = 200, *Z* = −5.05, *P* < 0.001*
Lipase (IU/l)			
(77–589)	321 (236–412.5)	208 (125–390)	*U* = 799.5, *Z* = 1.70, *P* = 0.087
Sodium (meq/l)			
(144–150)	147.5 ± 1.61	146.4 ± 2.35	*t* = 2.32, *df* = 70, *P* = 0.02*
Urea (mg/dl)			
(20–48)	33 (27–38.5)	23 (22–39)	*U* = 674.5, *Z* = 0.29, *P* = 0.761
Cr (mg/dl)			
(0.7–1.4)	1.11 (1.02–1.25)	0.85 (0.72–1.22)	*U* = 833, *Z* = 2.65, *P* = 0.008*
SDMA (μg/dl)			
(0–15)	8.40 (7.55–11.65)	13.50 (11–17)	*U* = 218, *Z* = −4.84, *P* < 0.001*

Data are expressed as mean ± standard deviation or median and interquartile range

*WBC* white blood cell concentration, *CRP* C-reactive protein, *PON-1* paraoxonase-1, *Ft* ferritin, *Hp* haptoglobin, *TIBC* total iron binding capacity, *Alb* albumin, *Glob* globulins, *GGT* gamma-glutamyl transferase, *Cr* creatinine, *SDMA* symmetric-dimethylarginine, *U* Mann–Whitney *U*-test, *t* *t*-test, *df* degrees of freedom, *Z* Mann–Whitney *Z*-score

^*^Statistically significant differences between healthy dogs and dogs with leishmaniosis

**Table 3:** Urinalysis and urinary chemistry of healthy dogs and dogs with leishmaniosis

Parameter	Healthy dogs	Dogs with leishmaniosis	Statistical analysis
(Reference interval)	*n* = 35	*n* = 37	
USG			
(1015–1050)	1042 ± 13.72	1033 ± 13.53	*t*_(70)_ = 2.38, *P* = 0.020*
UPC			
(0.1–0.5)	0.2 (0.15–0.2)	0.8 (0.3–4.90)	*U* = 97, *Z* = −6.21, *P* < 0.001*
uAm/Cr			
(0.1–50)	0.7 (0.4–1.10)	133.7 (7.7–1200)	*U* = 103.5, *Z* = −6.13, *P* < 0.001*
uFerr/Cr			
(0–25)	1 (0–3)	28 (8–42)	*U* = 132, *Z* = −5.81, *P* < 0.001*
uGGT/Cr			
(13–22)	21 (4–62.5)	50 (25.6–92.8)	*U* = 171, *Z* = −5.37, *P* < 0.001*
uGluc/Cr			
(2–8.5)	3.10 (2.75–3.60)	4.9 (4.2–7)	*U* = 237.5, *Z* = −4.62, *P* < 0.001*
FeNa (%)			
(0.1–1)	0.28 (0.21–0.54)	0.30 (0.17–0.63)	*U* = 633.5, *Z* = −0.16, *P* = 0.892
uCr (mg/dl)			
(125–324)	233 (161–304.5)	122 (79–207)	*U* = 964, *Z* = 3.56, *P* < 0.001*
Urinary podocin (ng/ml)	15.1 (11.75–17.87)	8.63 (7.08–13.56)	*U* = 984.5, *Z* = 3.79, *P* < 0.001*
Urinary nephrin (ng/ml)	3.2 (3.62–5.43)	2.67 (2.06–3.44)	*U* = 890, *Z* = 2.73, *P* = 0.006*
uPoC × 10^–6^	6.4 (5.5–8.6)	7.5 (5.3–10.1)	*U* = 569, *Z* = −0.89, *P* = 0.376
uNeC × 10^–6^	1.6 (1.1–2.1)	2.0 (1.1–2.8)	*U* = 528, *Z* = −1.35, *P* = 0.178

Data are expressed as mean ± standard deviation or median and interquartile range

*USG* urine-specific gravity, *UPC* urine protein-to-creatinine ratio, *uAm/Cr* urinary amylase-to-creatinine ratio, *uFerr/Cr* urinary ferritin-to-creatinine ratio, *uGGT* urinary gamma-glutamyltransferase, *uGlu/Cr* urinary glucose-to-creatinine ratio, *FeNa* fractional excretion of sodium, *uCr* urinary creatinine, *uPoC* urinary podocin-to-creatinine ratio, *NeC* urinary nephrin-to-creatinine ratio, *t* *t*-test, *df* degrees of freedom, *U* Mann–Whitney *U*-test; *Z* Mann–Whitney *Z*-score

^*^Statistically significant differences between healthy dogs and dogs with leishmaniosis

There was a significant increase of CRP, Ft, Hp, Glob, amylase, and SDMA, and a significant decrease of PON-1, iron, TIBC, and Alb in dogs with leishmaniosis compared with healthy dogs [Mann–Whitney *U*-test, *U* = 163, *Z* = −5.46, *P* < 0.001; *U* = 111.5, *Z* = −6.04, *P* < 0.001; *U* = 129.5, *Z* = −5.84, *P* < 0.001; *t*-test, *t*_(38)_ = −6.84, *P* < 0.001; *U* = 200, *Z* = −5.05, *P* < 0.001; *U* = 218, *Z* = −4.84, *P* < 0.001; *U* = 833.5, *Z* = 2.09, *P* = 0.036; *U* = 1038, *Z* = 4.39, *P* < 0.001; *U* = 1006.5, *Z* = 4.04, *P* < 0.001, *U* = 1107.5, *Z* = 5.18, *P* < 0.001, respectively, Table 2].

There was no statistical difference in urea between healthy dogs and dogs with leishmaniosis, but the latter had a significant lower Cr concentration and higher SDMA compared with healthy dogs (*U* = 674.5, *Z* = 0.29, *P* = 0.761; *U* = 883, *Z* = 2.65, *P* = 0.008; *U* = 218, *Z* = −4.84, *P* < 0.001, respectively, Table 2).

Compared with healthy dogs, dogs with leishmaniosis had significantly lower USG and uCr and increased UPC, uAm/Cr, uFerr/Cr, uGGT/Cr, uGlu/Cr [*t*_(70)_ = 2.38, *P* = 0.02; *U* = 964, *Z* = 3.56, *P* < 0.001; *U* = 97, *Z* = −6.21, *P* < 0.001; *U* = 103.5, *Z* = −6.13, *P* < 0.001; *U* = 132, *Z* = −5.81, *P* < 0.001; *U* = 171, *Z* = −5.37, *P* < 0.001; *U* = 237.5, *Z* = −4.62, *P* < 0.001, respectively, Table 3]. Urinary podocin and nephrin concentrations were significantly lower in dogs with leishmaniosis compared with healthy dogs, but there were no statistical differences in the uPoC and uNeC between the two groups (*U* = 984.5, *Z* = 3.79, *P* < 0.001; *U* = 890, *Z* = 2.73, *P* = 0.006; *U* = 569, *Z* = −0.89, *P* = 0.376; *U* = 528, *Z* = −1.35, *P* = 0.178, respectively, Table 3).

Renal ultrasound was performed in 35 dogs with leishmaniosis, and the more common ultrasonographic changes were increased renal cortical and/or medullary echogenicity in 18/35 dogs and increased renal cortical and/or medullary echogenicity and decreased renal corticomedullary distinction in 11/35 dogs.

According to the IRIS stage, dogs were classified as: stage I (*n* = 30, 18 were proteinuric and 12 non proteinuric), stage II (*n* = 4, 2 proteinuric and 2 non proteinuric), stage III (*n* = 1, proteinuric), and stage IV (*n* = 2, both proteinuric), and subsequently aggregated as stage I (*n* = 30) and as stages II + III + IV (*n* = 7) for statistical analysis. According to the IRIS staging used, urea, Cr, SDMA, and various urinary parameters are reported in healthy dogs and dogs with leishmaniosis in Table 4. Creatinine was significantly decreased in IRIS stage I compared with healthy dogs and with dogs in IRIS stage II + III + IV [analysis of variance (ANOVA), *F*_(2, 69)_ = 54.77, *P* < 0.0001, post hoc, *t* (*) = −5.64, *df* = 63, *P* < 0.001; post hoc, *t* (°) = −7.52, *df* = 40, *P* < 0.001, respectively, Table 4]. There was a significant increase in SDMA in dogs in IRIS stage II + III + IV compared with healthy dogs and with dogs in IRIS stage I [*F*_(2, 69)_ = 54.77, *P* < 0.0001, post hoc, *t* (^) = −4.91, *df* = 63, *P* < 0.001; post hoc, *t* (°) = − 8.47, *df* = 40, *P* < 0.001, respectively, Table 4]. A significant increase in UPC was observed in dogs in IRIS stage II + III + IV compared with healthy dogs and with dogs in IRIS stage I [Kruskal–Wallis *H*-test, *H* = 43.64, *df* = 2, *P* < 0.0001, post hoc, *U* (^) = 3.5, *Z* = −8.16, *P* < 0.001; post hoc*, U* (°) = 34, *Z* = −6.84, *P* = 0.006, respectively, Table 4]. The uAm/Cr was significantly higher in dogs in IRIS stage II + III + IV compared with healthy dogs and with dogs in IRIS stage I [*H* = 40.71, *df* = 2, *P* < 0.0001, post hoc *U* (^) = 0, *Z* = −8.28, *P* < 0.001; post hoc *U* (°) = 37, *Z* = −6.37, *P* = 0.007, respectively, Table 4]. Dogs in IRIS stage I and IRIS stage II + III + IV had significantly higher uFerr/Cr compared with healthy dogs [*H* = 35.88, *df* = 2, *P* < 0.0001, post hoc *U* (*) = 130.5, *Z* = −12.10, *P* < 0.001; post hoc *U* (^) = 1.5, *Z* = −8.23, *P* < 0.001, respectively, Table 4]. The uGGt/Cr was higher in dogs in IRIS stage I and IRIS stage II + III + IV compared with healthy dogs [*H* = 29.51, *df* = 2, *P* < 0.0001, post hoc *U* (*) = 161, *Z* = − 11.70, *P* < 0.001; post hoc *U* (^) = 10, *Z* = −7.95, *P* < 0.001, respectively, Table 4].

**Table 4:** Various serum and urinary parameters of healthy dogs and dogs with leishmaniosis based on IRIS staging

Parameters	Healthy dogs	IRIS stage I dogs	IRIS stages II-III-IV dogs	Statistical analysis	Post hoc
(Reference interval)	*n* = 35	*n* = 30	*n* = 7		
Urea (mg/dl)					
(20–48)	33 (27–38.5)	25.5 (20.25–36.75)	129 (85.5–181.0)	*H* = 18.46, *df* = 2 *P* < 0.001	*H* (^) = 10,* P* < 0.001; *H* (°) = *10, P* < 0.001
Cr (mg/dl)					
(0.7–1.4)	1.11 ± 0.17	0.83 ± 0.23	3.16 ± 1.65	*F* = 57.19, *df* = 2.69, *P* < 0.001	*t* (*) = 5.64, *df* = 63,* P* < 0.001; *t* (^) = −7.52, *df* = 40, *P* < 0.001; *t* (°) = −7.77*, df* = 35,* P* < 0.001
SDMA (μg/dl)					
(0 -15)	9.13 ± 2.91	12.7 ± 2.94	33.24 ± 16.33	*F* = 54.77, *df* = 2.69, *P* < 0.001	*t* (*) = −4.91, *df* = 63, *P* < 0.001; *t* (^) = −8.47, *df* = 40, *P* < 0.001; *t* (°) = −6.72*, df* = 35,* P* < 0.001
USG					
(1015–1050)	1042 ± 13.72	1035.2 ± 13.26	1028.7 ± 14.42	*F* = 0.04, *df* = 2.69, *P* = 0.036	*t* (^) = 2.26, *df* = 40, *P* = 0.029
UPC					
(0.1–0.5)	0.2 (0.15–0.20)	0.60 (0.30–1.57)	9.7 (4.9–14.1)	*H* = 43.6, *df* = 2 *P* < 0.001	*U* (*) = 93.5, *Z* = −6.24, *P* < 0.001;* U* (^) = 3.5, *Z* = −8.16, *P* < 0.001;* U* (°) = 34, *Z* = −6.84, *P* = 0.006
uAm/Cr					
(0.1–5)	0.7 (0.4–1.10)	89.15 (4.02–364.0)	1401.3 (950.4–1752.8)	*H* = 40.7, *df* = 2 *P* < 0.001	*U* (*) = 103.5,* Z* = 12.5, *P* < 0.001;* U* (^) = 0, *Z* = −8.28, *P* < 0.001;* U* (°) = 37, *Z* = −6.73, *P* = 0.007
uFerr/Cr					
(0–25)	1 (0–3)	23.5 (6.25–40.75)	31 (28.5–53)	*H* = 35.9, *df* = 2 *P* < 0.001	*U* (*) = 130.5,* Z* = −12.10, *P* < 0.001;* U* (^) = 1.5,* Z* = −8.23, *P* < 0.001
uGGT/Cr					
(13–22)	15.7 (12.15–19.7)	45.25 (23.52–93.3)	78.7 (49–88.45)	*H* = 29.5, *df* = 2 *P* < 0.001	*U* (*) = 161,* Z* = −11.70, *P* < 0.001;* U* (^) = 10, *Z* = −7.95, *P* < 0.001
uGluc/Cr					
(2–8.5)	3.10 (2.75–3.60)	4.9 (4.23–6.65)	5.1 (3.4–13.6)	*H* = 21.4, *df* = 2 *P* < 0.001	*U* (*) = 165.5,* Z* = 11.6, *P* < 0.001
FeNa (%)					
(0.1–1)	0.28 (0.21–0.54)	0.29 (0.16–0.61)	0.49 (0.32–1.36)	*H* = 21.4, *df* = 2 *P* = 0.426	
uCr (mg/dl)					
(125–324)	233 (161–304.5)	147.5 (82.25–206.8)	127 (79.5–220.5)	*H* = 12.8, *df* = 2 *P* = 0.0017	*U* (*) = 780.5,* Z* = −3.55, *P* < 0.001

Data are expressed as mean ± standard deviation or median and interquartile range

*Cr* creatinine, *SDMA* symmetric dimethylarginine, *USG* urine-specific gravity, *UPC* urine protein-to-creatinine ratio, *uAm/Cr* urinary amylase-to-creatinine ratio, *uFerr/Cr* urinary ferritin-to-creatinine ratio, *uGGT* urinary gamma-glutamyltransferase, *uGlu/Cr* urinary glucose-to-creatinine ratio, *FeNa* fractional excretion of sodium, *uCr* urinary creatinine, *H* Kruskall–Wallis *H*-test, *df* degrees of freedom, *F* Fisher’s exact test, *t* *t*-test, *U* Mann–Whitney *U*-test *Z* Mann–Whitney *Z*-score

^*^Statistically significant differences between healthy dogs and IRIS stage I dogs

^Statistically significant differences between healthy dogs and IRIS stages II + III + IV dogs

°Statistically significant differences between IRIS I and IRIS stage II + III + IV dogs

**Fig. 1:**
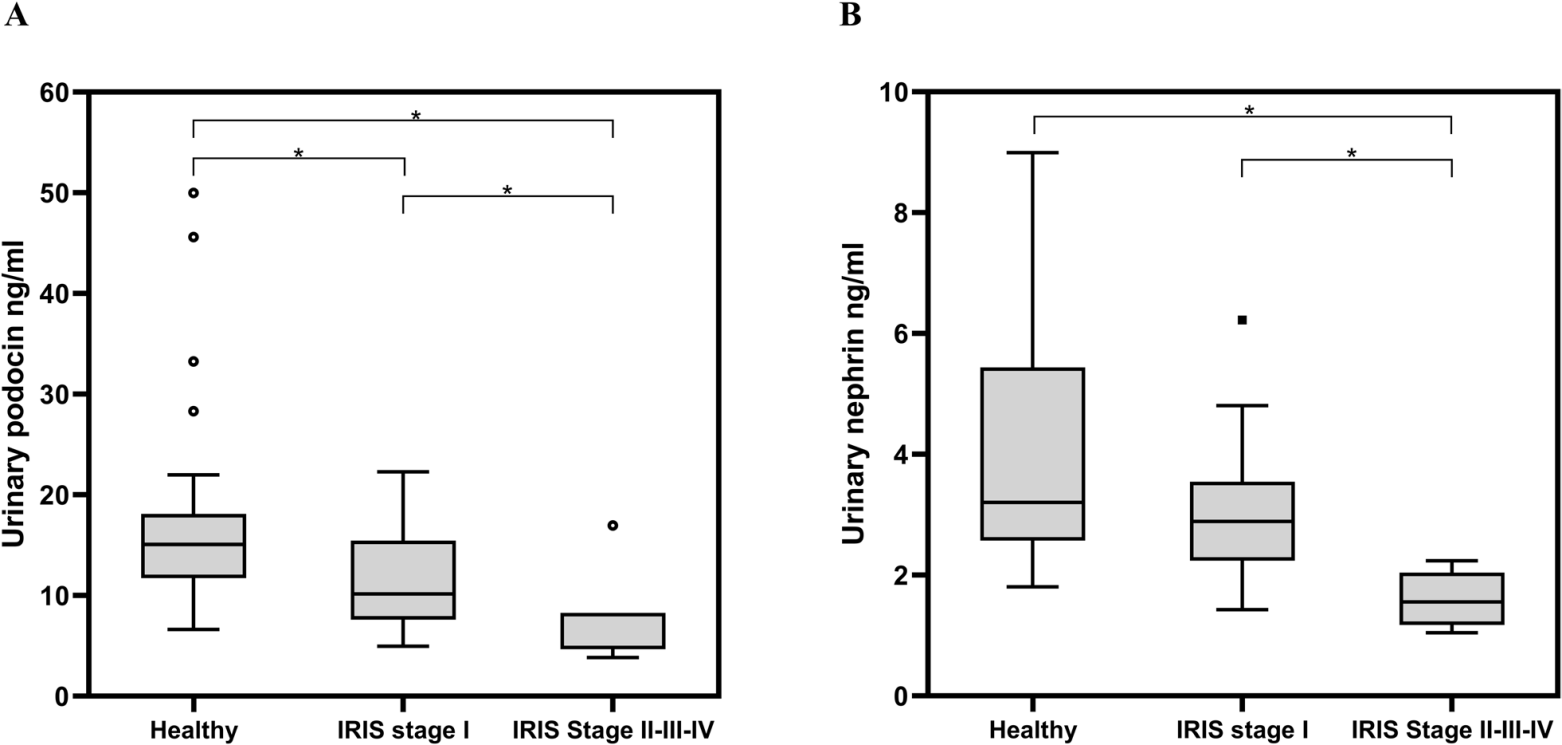
Box plots showing the concentrations of (**A**) urinary podocin and the concentrations of (**B**) nephrin in healthy dogs and dogs with leishmaniosis according to the IRIS staging. *Statistically significant difference between the median of each group

According to IRIS staging, higher urinary podocin and nephrin concentrations were observed in healthy dogs [median values and IQR 15.06 ng/ml (11.75–17.87) and 3.20 ng/ml (2.62–5.43), Fig. 1] compared with dogs in IRIS stages II + III + IV [median values and IQR 4.84 ng/ml (4.70–6.64) and 1.55 ng/ml (1.29–1.89); *H* = 18.42 and *H* = 17.97, *df* = 2, *P* = 0.0001, post hoc *U* (^) = 219, *Z* = −2.89, *P* = 0.001; post hoc *U* (^) = 237, *Z* = −3.01, *P* < 0.001, respectively, Fig. 1]. Urinary podocin and nephrin concentrations were significantly higher in dogs in IRIS stage I [median values and IQR 10.18 ng/ml (7.67–14.87) and 2.89 ng/ml (2.29–3.48), Fig. 1] compared with dogs in IRIS stages II + III + IV [post hoc *U* (°) = 172, *Z* = −2.49, *P* = 0.009; post hoc *U* (°) = 198, *Z* = − 3.71, *P* = 0.0001 respectively, Fig. 1]. Urinary podocin concentration was also higher in healthy dogs compared with dogs in IRIS stage I [median values and IQR 15.06 ng/ml (11.75–17.87) versus 10.18 ng/ml (7.67–14.87); post hoc *U* (*) = 765.5, *Z* = −3.75, *P* = 0.002, Fig. 1A] while urinary nephrin was similar in the two groups [*U* (*) = 653, *Z* = −1.69, *P* = 0.09, Fig. 1B].

No significant differences were found for the uPoC and uNeC between healthy dogs and dogs with leishmaniosis in different IRIS clinical stages (*H* = 4.83, *df* = 2, *P* = 0 0.089; *H* = 5.52, *df* = 2, *P* = 0.063, respectively, Table 5).

**Table 5:** The uPoC and uNeC in healthy dogs and dogs with leishmaniosis according to IRIS staging

Parameter	Healthy dogs	Stage I	Stages II + III + IV	Statistical analysis
	*n* = 35	*n* = 30	*n* = 7	
uPoC × 10^−6^	6.4	7.6	5.1	*H* = 4.83, *df* = 2, *P* = 0.089
	(5.5–8.6)	(5.9–10.1)	(3.3–6.8)	
uNeC × 10^−6^	1.6	2.1	1.1	*H* = 5.52, *df* = 2, *P* = 0.063
	(1.1–2.1)	(1.5–3.0)	(0.8–1.9)	

Data are expressed as median and interquartile range

*UPoC* urinary podocin-to-creatinine ratio, *uNeC* urinary nephrin-to-creatinine ratio, *H* Kruskall–Wallis *H*-test, *df* degrees of freedom

**Fig. 2:**
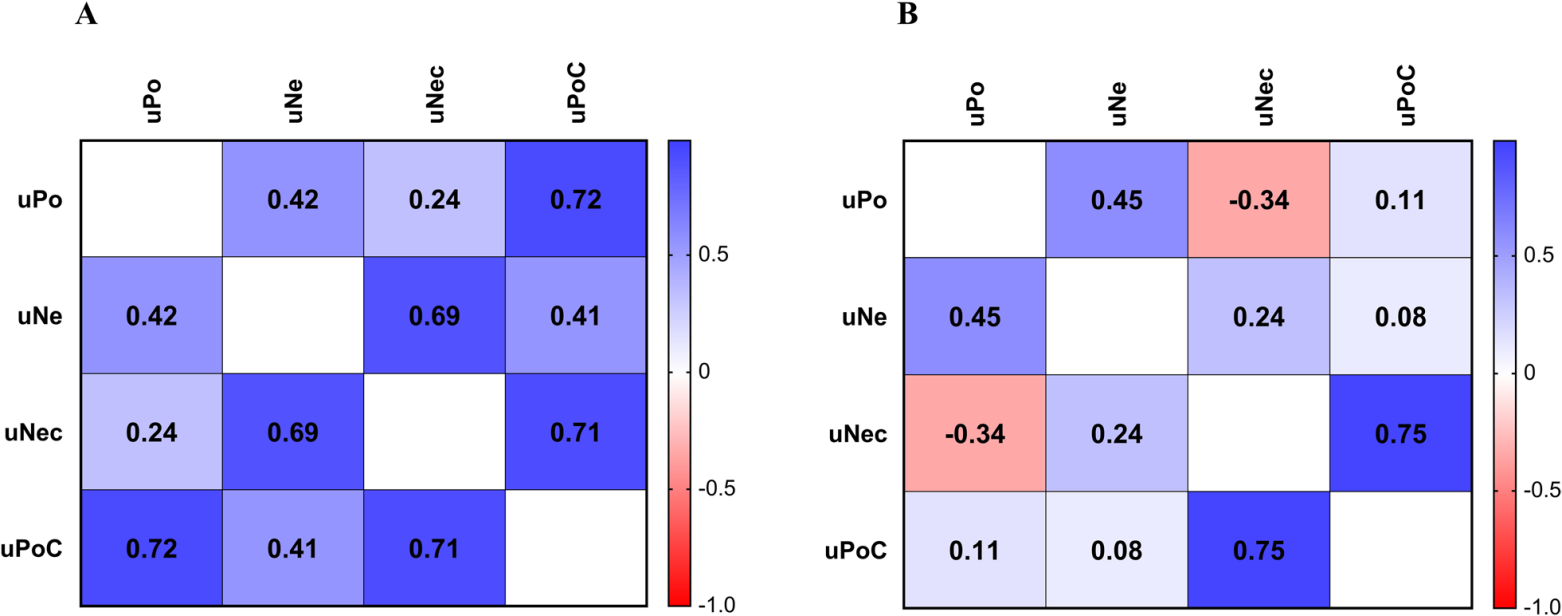
Correlation plot between uPo, uNe, uNeC, and uPoC in healthy dogs (**A**) and dogs with leishmaniosis (**B**). *uPo* urinary podocin concentration, *uNe* urinary nephrin concentration, *uNeC* urinary nephrin-to-creatinine ratio, *uPoC* urinary podocin-to-creatinine ratio

In healthy dogs and dogs with leishmaniosis, there was a moderate positive correlation between urinary podocin and urinary nephrin concentrations [Pearson’s coefficient correlation, *r*_(33)_ = 0.42, *P* = 0.01; *r*_(35)_ = 0.45, *P* = 0.005, respectively, Fig. 2A,B] and a strong positive correlation between uPoC and uNeC [*r*_(33)_ = 0.71 and *r*_(35)_ = 0.75, *P* < 0 0.0001, Fig. 2A,B]. In healthy dogs, there was a moderate correlation between urinary nephrin and uNeC and a strong correlation between urinary podocin and uPoC [*r*_(33)_ = 0.69 and *r*_(35)_ = 0.72; *P* < 0.0001, Fig. 2A,B]. According to IRIS staging, dogs in IRIS stage I had a moderate correlation between uPoC and uNeC [*r*_(28)_ = 0.67, *P* < 0.0001]. Dogs in IRIS stage II + III + IV showed a strong correlation between urinary podocin and urinary nephrin [*r*_(5)_ = 0.76, *P* = 0.04] and a very strong correlation between uPoC and uNeC [*r*_(5)_ = 0.96, *P* = 0.0006].

In dogs with leishmaniosis there was a moderate negative correlation between urinary podocin concentration and Cr, SDMA, UPC, and uAm/Cr [*r*_(35)_ = 0.40, *P* = 0.02; *r*_(35)_ = −0.53; *P* = 0.001; *r*_(35)_ = −0.52, *P* = 0.001; *r*_(35)_ = −0.54, *P* = 0.001, respectively, Table 6]. A strong positive correlation was observed between urinary podocin and USG in dogs with leishmaniosis [*r*_(35)_ = 0.74, *P* < 0.001, Table 6]. Urinary nephrin had a moderate negative correlation with Cr, SDMA, UPC, and uAm/Cr [*r*_(35)_ = −0.46, *P* = 0.004; *r*_(35)_ = −0.45, *P* = 0.006; *r*_(35)_ = −0.50, *P* = 0.002; *r*_(35)_  = −0.50, *P* = 0.002, respectively, Table 6]. According to IRIS staging, there was a moderate negative correlation between urinary podocin concentration and SDMA, UPC, and uAm/Cr in dogs in IRIS stage I [*r*_(28)_ = −0.58; *P* = 0.001; *r*_(28)_ = −0.43, *P* = 0.016; *r*_(28)_ = −0.44, *P* = 0.015, respectively, Table 7]. A strong positive correlation was observed between urinary podocin and USG in dogs in IRIS stage I and IRIS stages II + III + IV [*r*_(35)_ = 0.71, *P* < 0.001; *r*_(35)_ = 0.87, *P* = 0.01, respectively, Table 7]. A moderate negative correlation between urinary podocin and SDMA was found in dogs in IRIS stage I [*r*_(28)_ = −0.58; *P* = 0.001, Table 7]. A very strong correlation was found between urinary nephrin and USG in dogs in IRIS stage II + III + IV [*r*_(5)_ = 0.93, *P* = 0.002, Table 7].

**Table 6:** Correlations between urinary podocin and nephrin concentrations and various renal/urinary markers in the two groups

	Healthy dogs		Dogs with leishmaniosis	
	*n* = 35		*n* = 37	
Markers	*r*	*P*-value*	*r*	*P*-value*
uPo × Cr	0.21	0.22	**−0.40**	0.02*
uPo × SDMA	0	1.0	**−0.53**	0.001*
uPo × USG	0.29	0.1	**0.74**	< 0.001*
uPo × UPC	−0.05	0.77	**−0.52**	0.001*
uPo × uAm/Cr	0.34	0.04*	**−0.54**	0.001*
uPo × uFerr/Cr	0	0.99	−0.11	0.50
uPo × uGGT/cr	0.04	0.80	−0.06	0.70
uPo × uGlu/Cr	−0.14	0.42	−0.2	0.23
uNe × Cr	−0.09	0.50	**−0.46**	0.004*
uNe × SDMA	−0.17	0.76	**−0.45**	0.006*
uNe × USG	0.27	0.19	0.28	0.09
uNe × UPC	0.02	0.19	**−0.50**	0.002*
uNe × uAm/Cr	−0.18	0.64	**−0.50**	0.002*
uNe × uFerr/Cr	−0.10	0.36	−0.28	0.28
uNe × uGGT/Cr	−0.05	0.08	−0.09	0.61
uNe × uGlu/Cr	0.02	0.45	−0.22	0.19

^*^Pearson’s test, Bold: data considered significant *P* < 0.05 and *r* ≥ 0.4

*r* correlation coefficient, *uPo* urinary podocin concentration, *Cr* creatinine, *SDMA* symmetric-dimethylarginine, *USG* urine-specific gravity, *UPC* urine protein-to-creatinine ratio, *uAm/Cr* urinary amylase-to-creatinine ratio, *uFerr/Cr* urinary ferritin-to-creatinine ratio, *uGGT/Cr* urinary gamma-glutamyltransferase-to-creatinine ratio, *uGlu/Cr* urinary glucose-to-creatinine ratio, *uNe* urinary nephrin concentration

**Table 7:** Correlations between urinary podocin and nephrin concentrations and various renal/urinary markers according to IRIS stages

	IRIS stage I dogs		IRIS stage II-III-IV dogs		Dogs with leishmaniosis	
	*n* = 30		*n* = 7		*n* = 37	
Markers	*r*	*P*-value*	*r*	*P*-value*	*r*	*P*-value*
uPo × Cr	−0.07	0.73	−0.39	0.32	**−0.40**	0.02*
uPo × SDMA	**−0.58**	0.001*	**−0.71**	0.08	**−0.53**	0.001*
uPo × USG	**0.71**	< 0.001*	**0.87**	0.01*	**0.74**	< 0.001*
uPo × UPC	**−0.43**	0.016*	**−0.55**	0.20	**−0.52**	0.001*
uPo × uAm/Cr	**−0.44**	0.015*	**−0.59**	0.17	**−0.54**	0.001*
uPo × uFerr/Cr	−0.17	0.37	−0.01	0.98	−0.11	0.50
uPo × uGGT/cr	−0.03	0.86	**−0.50**	0.26	−0.06	0.70
uPo × uGlu/Cr	−0.12	0.53	−0.21	0.66	−0.2	0.23
uNe × Cr	0.05	0.79	**−0.51**	0.24	**−0.46**	0.004*
uNe × SDMA	0.01	0.96	**−0.53**	0.22	**−0.45**	0.006*
uNe × USG	0.16	0.41	**0.93**	0.002*	0.28	0.09
uNe × UPC	−0.36	0.06	−0.39	0.32	**−0.50**	0.002*
uNe × uAm/Cr	−0.37	0.05	−0.34	0.47	**−0.50**	0.002*
uNe × uFerr/Cr	−0.28	0.13	0.26	0.57	−0.28	0.28
uNe × uGGT/Cr	−0.10	0.58	0.03	0.94	−0.09	0.61
uNe × uGlu/Cr	−0.15	0.41	−0.07	0.88	−0.22	0.19

*r* correlation coefficient, *uPo* urinary podocin concentration, *Cr* creatinine, *SDMA* symmetric-dimethylarginine, *USG* urine-specific gravity, *UPC* urine protein-to-creatinine ratio, *uAm/Cr* urinary amylase-to-creatinine ratio, *uFerr/Cr* urinary ferritin-to-creatinine ratio, *uGGT/Cr* urinary gamma-glutamyltransferase-to-creatinine ratio, *uGlu/Cr* urinary glucose-to-creatinine ratio, *uNe* urinary nephrin concentration

^*^Pearson’s test, Bold: data considered significant *P* < 0.05 and *r* ≥ 0.4

In dogs with leishmaniosis there was a moderate negative correlation between uNeC and USG (*r*_(35)_ = −0.54, *P* = 0.001, Table 8). According to IRIS staging, in dogs in IRIs stage I a moderate correlation was found between uNeC and SDMA and USG [*r*_(28)_ = 0.52, *P* = 0.003; *r*_(28)_ = −0.64, *P* < 0.001, Table 9], and a strong correlation between uNeC and UPC and uNeC and uAm/Cr [*r*_(28)_ = 0.84, *P* = 0.02; *r*_(28)_ = 0.80, *P* = 0.03, respectively, Table 9].

**Table 8:** Correlations between the uNeC and uPoC and various renal/urinary markers in the two groups

	Healthy dogs		Dogs with leishmaniosis	
	*n* = 35		*n* = 37	
Markers	*r*	*P*-value*	*r*	*P*-value*
uPoC × Cr	−0.12	0.50	−0.29	0.08
uPoC × SDMA	−0.05	0.76	−0.25	0.14
uPoC × USG	−0.22	0.19	−0.24	0.15
uPoC × UPC	0.23	0.19	0.18	0.30
uPoC × uAm/Cr	−0.08	0.64	0.19	0.27
uPoC × uFerr/Cr	0.16	0.36	0.21	0.20
uPoC × uGGT/Cr	0.30	0.08	0.18	0.27
uPoC × uGlu/Cr	0.13	0.45	0.11	0.50
uNeC × Cr	−0.35	0.04*	−0.21	0.21
uNeC × SDMA	−0.1	0.55	−0.05	0.75
uNeC × USG	−0.29	0.08	**−0.54**	0.001*
uNeC × UPC	0.29	0.10	0.21	0.20
uNeC × uAm/Cr	0.1	0.57	0.26	0.12
uNeC × uFerr/Cr	0.03	0.85	0.22	0.19
uNeC × GGT/cr	0.2	0.25	0.16	0.36
uNeC x Glu/Cr	0.3	0.08	0.11	0.53

*r* correlation coefficient, *uPoC* urinary podocin-to-creatinine ratio, *Cr* creatinine, *SDMA* symmetric-dimethylarginine, *USG* urine-specific gravity, *UPC* urine protein-to-creatinine ratio, *uAm/Cr* urinary amylase-to-creatinine ratio, *uFerr/Cr* urinary ferritin-to-creatinine ratio, *uGGT/Cr* urinary gamma-glutamyltransferase-to-creatinine ratio, *uGlu/Cr* urinary glucose-to-creatinine ratio, *uNeC* urinary nephrin-to-creatinine ratio

^*^Pearson’s test, Bold: data considered significant *P* < 0.05 and *r* ≥ 0.4

**Table 9:** Correlations between the uNeC and uPoC and various renal/urinary markers according to IRIS stages

	IRIS stage I dogs		IRIS stage II–III–IV dogs		Dogs with leishmaniosis	
	*n* = 30		*n* = 7		*n* = 37	
Markers	*r*	*P*-value*	*r*	*P*-value*	*r*	*P*-value*
uPoC × Cr	−0.35	0.06	−0.22	0.64	−0.29	0.08
uPoC × SDMA	0.08	0.69	−0.28	0.55	−0.25	0.14
uPoC × USG	−0.28	0.13	−0.34	0.46	−0.24	0.15
uPoC × UPC	0.06	0.74	**0.70**	0.08	0.18	0.30
uPoC × uAm/Cr	0.14	0.45	**0.67**	0.1	0.19	0.27
uPoC × uFerr/Cr	0.25	0.18	0.08	0.86	0.21	0.20
uPoC × uGGT/Cr	0.25	0.18	−0.04	0.94	0.18	0.27
uPoC × uGlu/Cr	0.39	0.04*	0.26	0.58	0.11	0.50
uNeC × Cr	−0.14	0.45	−0.11	0.81	−0.21	0.21
uNeC × SDMA	**0.52**	0.003*	−0.06	0.90	−0.05	0.75
uNeC × USG	**−0.64**	< 0.001*	**−0.47**	0.29	**−0.54**	0.001*
uNeC × UPC	0.22	0.24	**0.84**	0.02*	0.21	0.20
uNeC × uAm/Cr	0.28	0.13	**0.80**	0.03*	0.26	0.12
uNeC × uFerr/Cr	0.23	0.23	0.12	0.80	0.22	0.19
uNeC × GGT/cr	0.17	0.38	0.17	0.72	0.16	0.36
uNeC × Glu/Cr	0.3	0.03*	0.33	0.48	0.11	0.53

*r* correlation coefficient, *uPoC* urinary podocin-to-creatinine ratio, *Cr* creatinine, *SDMA* symmetric-dimethylarginine, *USG* urine-specific gravity, *UPC* urine protein-to-creatinine ratio, *uAm/Cr* urinary amylase-to-creatinine ratio, *uFerr/Cr* urinary ferritin-to-creatinine ratio, *uGGT/Cr* urinary gamma-glutamyltransferase-to-creatinine ratio, *uGlu/Cr* urinary glucose-to-creatinine ratio, *uNeC* urinary nephrin-to-creatinine ratio

^*^Pearson’s test, Bold: data considered significant *P* < 0.05 and *r* ≥ 0.4

No correlation was found in leishmaniotic dogs between SBP and UPC, urinary podocin, urinary nephrin, uPoC, and uNeC [*r*_(35)_ = 0.20, *P* = 0.25; *r*_(35)_ = −0.31, *P* = 0.06; *r*_(35)_ = −0.18, *P* = 0.29; *r*_(35)_ = −0.09, *P* = 0.58; *r*_(35)_ = −0.32, *P* = 0.05, respectively].

On the basis of multivariable logistic regression, leishmaniosis was associated with increase on SDMA [odds ratio (OR) 1.4, 95% CI 1.08–1.92; *Z* = 0.34, *P* = 0.015), and uAm/Cr (OR 1.7, 95% CI 1.15–3.09; *Z* = 0.53, *P* = 0.04]. The area under the curve (AUC) of the model was 0.95 (95% CI 0.89–0.99) and the Hosmer–Lemeshow goodness-of-fit test validated the model [*χ*-squared chi-squared test, *χ*^2^ = 3.56, *df* = 6, *P* = 0.74].

## Discussion

In the present study, podocin and nephrin were evaluated in the urine of healthy dogs and dogs with leishmaniosis. This is the first report in which urinary podocin and nephrin were detected in dogs affected by leishmaniosis as markers of podocyturia. Podocin was detected in the urine of all healthy dogs using the same commercial ELISA test as previously documented [23], while nephrin was measured for the first time in the urine of healthy dogs using a commercial ELISA test. That result contrasts with those of some human studies in which nephrin measured with this methodology was not detected in the urine of healthy controls [43, 47]. In a veterinary study, nephrin mRNA was measured in urine sediment from healthy dogs with a positive detection rate of 40% [24]. It should be kept in mind that the use of different methods, which vary in terms of sensitivity, makes it difficult to compare the results obtained from different studies, and the presence of physiological podocyturia in dogs remains under discussion [48].

Interestingly, urinary podocin and nephrin concentrations were lower in dogs with leishmaniosis compared with healthy dogs. These decreases are difficult to interpret due to the heterogeneity of the dogs included in the present study. Dogs with leishmaniosis belonged to different stages of IRIS, meaning a different degree of disease severity and of renal involvement. When dogs were classified according to the IRIS staging, urinary podocin and nephrin concentrations were significantly lower in dogs in IRIS stages II + III + IV compared with dogs in IRIS stage I and with healthy dogs. These findings are in part consistent with those of dogs with chronic renal disease (without an etiological diagnosis of their renal disease) in IRIS stages III and IV, in which a significant decrease has been described in the presence of podocin and nephrin mRNA in urine [24]. The results of the latter study were consistent with the reduction in the number of podocytes in the kidneys that has previously been identified in dogs with chronic kidney disease at various stages but without an etiological diagnosis [49].

Dogs in stage I of IRIS had lower and similar urinary podocin and nephrin concentrations compared with healthy dogs, respectively. These results were totally unexpected, difficult to explain, and contrasted with the higher detection of urinary podocin and nephrin mRNA levels in dogs with chronic kidney disease in IRIS stages I and II [24]. In the study by de Souza et al. [24], all dogs in stages IRIS I and II had proteinuria, while in the current study, some dogs in stage I had proteinuria and some did not. It is possible that proteinuria was the cause of the variations between the two investigations. Proteinuria is considered a hallmark of glomerular disease [50], but the absence of proteinuria does not rule out the presence of glomerular damage, as shown in the study by Costa et al. [6] in which non azotemic and non proteinuric dogs with leishmaniosis had on renal histopathology minor glomerular abnormalities and mesangial proliferative glomerulonephritis. In the current study, the lack of a kidney biopsy does not allow for confirmation or exclusion of glomerular damage in non-proteinuric dogs in IRIS stage I with leishmaniosis.

When CanL causes kidney involvement, renal disease is mainly of glomerular origin with different histopathological forms of glomerulonephritis [6, 7, 51] that can progress to tubulointerstitial lesions, azotemia, and ultimately to end-stage renal failure [9, 10]. Several human studies have reported that podocyte loss was correlated with the development of glomerulopathy [52], and podocyturia could be the first indicator of kidney failure in dogs due to the damage of the glomerular basement membrane [21] and the onset of proteinuria [30]. Proteinuria is the main clinical pathological finding of glomerular disease in CanL [9] which varies in severity in the different forms of glomerulonephritis. In humans, the rate of excretion of podocytes reflects the type of disease and disease activity (with active renal disease defined by a urinary albumin/creatinine ratio > 300 µg/mg) [22]. Podocyturia has been shown to be positively correlated with active renal disease, but not with inactive one [22, 53, 54]. In dogs, proteinuria is generally considered a marker of kidney progression when combined with azotemia [55]. In the present study, most dogs with leishmaniosis were not azotemic and had a variable degree of proteinuria. Furthermore, no kidney biopsy was performed in dogs with leishmaniosis, so on the one hand, a final diagnosis of the type of renal disease was missing, and on the other hand, it is not known in which dogs an active or inactive renal disease was present. The definition of active/inactive renal disease has not been well established in veterinary medicine. De Souza and colleagues have shown that dogs with chronic kidney disease in stages I and II had more frequent detection of urinary nephrin and podocin m-RNA, and this result combined with high proteinuria could suggest active kidney injury [24]. More studies are necessary to better understand which mechanisms intervene during renal disease in CanL.

No significant differences were observed between dogs with leishmaniosis and healthy dogs in the uPoC and uNeC. However, IRIS stage I dogs showed an increasing trend compared with healthy dogs, and with IRIS stages II + III + IV dogs a decreasing trend. These last results seemed in line with those of a study in which dogs in IRIS stages I and II had increased podocin and nephrin mRNA expression and, dogs in IRIS stages III and IV decreased expression of both markers [24]. In contrast, in a study of dogs with degenerative mitral valve disease, a control group of dogs with kidney disease (of unknown origin) had a significantly increased uPoC compared with healthy dogs, but the IRIS stage they belonged to was not considered [23].

In the current study, dogs of both groups (healthy versus leishmaniotic) had a positive correlation between urinary podocin and nephrin and uPoC and uNeC. These results agree with those of humans with diabetes mellitus in which a strong positive correlation between podocin and nephrin mRNA in urine was found, suggesting a common pathophysiological pathway for their presence in urine [56]. Podocytes cover the glomerulus, and their adjacent foot processes form a principal barrier called the slit diaphragm. Two essential parts of the slit diaphragm are podocin and nephrin [57], which interact directly when podocin binds to nephrin’s cytoplasmic tail and activates it [58]. On the basis of the results in the present study and the anatomical–functional link between podocin and nephrin, both proteins could be used as markers of podocyturia in dogs. Interestingly, there was a positive correlation between urinary podocin and uPoC, and urinary nephrin and uNeC in healthy dogs, but not in dogs with leishmaniosis (confirmed in both IRIS stage I and in IRIS stage II + III + IV dogs). A possible explanation must be sought in the lower uCr concentration and lower USG in dogs with leishmaniosis compared with healthy dogs. In this study, all dogs with leishmaniosis had kidney disease even if the degree of kidney disease varies according to IRIS staging.

In dogs with leishmaniosis, urinary podocin and nephrin concentrations were negatively correlated with Cr, SDMA, and UPC. The negative correlation between urinary podocin and nephrin and Cr and SDMA could be explained by the fact that with the progression of renal disease, there is a reduction in the number of nephrons and podocytes that adhere to the glomerular basement membrane [49]. When uPoC and uNeC were considered, the correlation with Cr and SDMA was not significant (except for uNeC and SDMA in dogs in IRIS stage I), perhaps due to the different values of uCr in the group of dogs with leishmaniosis and the low number of dogs in each stage (according to the IRIS staging). The negative correlation between urinary podocin and nephrin concentrations and UPC is difficult to explain given that proteinuria was found in both IRIS stage I and IRIS stages II + III + IV dogs, although proteinuria was higher in IRIS stages II + III + IV dogs. In line with the current study, a prior study found that dogs with advanced stages of chronic renal disease had higher levels of proteinuria and lower levels of podocin and nephrin mRNA in urine sediment. This was likely caused by a decrease in podocyte population [49] and the contribution of podocytopenia to proteinuria [24]. Interestingly, in a human study, the authors showed that the association between podocyturia and proteinuria varied according to the type of glomerular disease: a high correlation in minimal change disease and a low correlation in membranous nephropathy [59]. Urinary podocin was positively correlated with USG in dogs with leishmaniosis. There was a positive correlation between urinary nephrin and USG in dogs in IRIS stage II + III + IV but not in dogs in IRIS stage I. In a human study, podocyturia has been shown to be higher in more concentrated urine, but the mechanism remains to be defined [22].

In the present study, dogs with leishmaniosis had a significant inflammatory response compared with healthy dogs. These results are in agreement with the increase in acute phase proteins such as CRP, Ft, and Hp [60, 61] and the decrease in iron and TIBC [62], as previously described. All these findings suggest that the parasite triggers an acute phase response in the host that changes iron status [60, 62].

Although dogs with leishmaniosis had overall a lower serum Cr concentration (with a Cr within the normal reference interval in dogs in IRIS stage I and increased in IRIS stage II + III + IV) compared with healthy dogs, SDMA was higher in the group of sick dogs (even if the majority of dogs were in the normal reference interval). No clear explanation was found for the lower Cr concentration in dogs in IRIS stage I compared with healthy dogs. Apparently, no muscle waist was appreciated on physical examination, but different owners reported weight loss in the last month as one of the main clinical signs. It is possible that there was a decreased muscle mass, which influences Cr but not SDMA concentrations [63, 64]. Only the body condition score and not the muscle condition score was evaluated in the groups, and therefore is difficult to interpret correctly this difference in a clinical context. Another aspect to consider is the intraindividual variability of SDMA [63] and the potential limitation of the SDMA assay [65].

Proteinuria, and different markers of tubular and glomerular injury, were measured, and interestingly, UPC, uFerr/Cr, and uGGT/Cr were significantly increased in dogs with leishmaniosis compared with healthy dogs, highlighting that during this disease process there can be tubular and/or glomerular damage even before the onset of kidney dysfunction as shown by other authors [10, 19, 66, 67].

The uAm/Cr was significantly increased in dogs with leishmaniosis compared with healthy dogs and according to statistical analysis resulted likely as marker of renal damage during this infection. On the basis of its molecular weight of 54 kD [68, 69], it has purposed as a marker of tubular and/or glomerular injury. In this sense, Schepper et al. stated that in the absence of pancreatic disease, amylasuria could be an indicator of renal glomerular disease in female dogs with pyometra [70].

This study has several limitations. The limited number of dogs studied could have limited the statistical power to detect significant differences in the variables studied (especially when the dogs were further divided into different groups according to the IRIS staging). Another limitation can be the division of dogs in IRIS stage I and IRIS stage II + III + IV, which include dogs with a very wide range of kidney dysfunction. The lack of a kidney biopsy in dogs with leishmaniosis does not allow for determining whether histopathological changes occurred in the kidneys and the eventual association between a specific type of renal pathological findings with urinary podocin and nephrin concentrations. Another aspect to consider is the fact that a normal reference interval with an adequate number of healthy dogs (at least 120 healthy dogs) for urinary podocin and nephrin concentrations and for uPoC and uNeC has not yet been established in veterinary medicine. Urinary podocin and nephrin concentrations are dependent on urine concentration, and therefore, it is recommended to normalize them to uCr concentration. It remains to be defined whether this criterion should be applied in hypersthenuria urine (dogs with a USG > 1030) and in the case of using urinary cellular sediment rather than urinary supernatant [71]. In veterinary medicine, only one study in dogs described the use of urinary podocin normalized to uCr concentration [23, 72]. Urinary podocin has been measured with different methodologies in human medicine without defining which should be the gold standard, and in a study in which urinary podocin was quantified by an ELISA test the result was normalized to uCr concentration [72]. In human literature, the most commonly used technique for measuring urine nephrin is an ELISA test, but there is no consensus on how to report urinary nephrin concentration [73]. As reviewed by Mesfine et al., urinary nephrin concentration has been mostly reported in ng/ml and a few times as urinary nephrin concentration corrected for uCr concentration without any comparison between these two ways [73]. More research is needed to understand whether urinary podocin and nephrin concentrations can be useful in clinical practice for the early detection of glomerular injury in CanL and other canine renal diseases.

## Conclusions

Urinary podocin and nephrin concentrations can be measured using a commercial ELISA test in healthy dogs and dogs with leishmaniosis. Dogs with leishmaniosis appeared to have a low concentration of podocin and nephrin in more advanced clinical stages of IRIS staging, where kidney disease was more severe compared with healthy dogs and dogs with mild disease. The results of this study suggest that urinary podocin and nephrin are not good markers for early diagnose of renal disease in dogs with leishmaniosis.

## Supplementary Information

Additional file 1.

## Data Availability

All data supporting the main conclusions are available in the manuscript and its associated files.

## Abbreviations

CanL: Canine leishmaniosis
*L. infantum*: *Leishmania infantum*
ELISA: Enzyme-linked immunosorbent assay
IRIS: International Renal Interest Society
CBC: Complete blood count
q-PCR: Real-time polymerase chain reaction
SBP: Systolic blood pressure
WBC: White blood cell count
PON-1: Paraoxonase-1
Hp: Haptoglobin
Ft: Ferritin
CRP: C-reactive protein
TIBC: Total iron-binding capacity
Alb: Albumin
Glob: Globulins
GGT: Gamma-glutamyl transferase
Cr: Creatinine
Gluc: Glucose
Na: Sodium
SDMA: Symmetric-dimethylarginine
USG: Urine-specific gravity
UPC: Urine protein-to-creatinine ratio
FeNa: Fractional excretion of sodium
uAm/Cr: Urinary amylase-to-creatinine ratio
uFerr/Cr: Urinary ferritin-to-creatinine ratio
uGGT/Cr: Urinary gamma-glutamyltransferase-to-creatinine ratio
uGlu/Cr: Urinary glucose-to-creatinine ratio
uCr: Urinary creatinine
uPoC: Urinary podocin-to-creatinine ratio
uNeC: Urinary nephrin-to-creatinine ratio
IQR: Interquartile range
ROC-AUC: Area under the receiver operating characteristic curve

## References

[1] Solano-Gallego L, Koutinas A, Miró G, Cardoso L, Pennisi MG, Ferrer L, et al. Directions for the diagnosis, clinical staging, treatment and prevention of canine leishmaniosis. Vet Parasitol. 2009;165:1–18. 19559536 10.1016/j.vetpar.2009.05.022

[2] LeishVet guidelines for the practical management of canine leishmaniosis—LeishVet. [cited 2024 Jun 10]. Available from: https://www.leishvet.org/publications/canine-leishmaniosis-guidelines/10.1186/1756-3305-4-86 PMC3125381 21599936

[3] Solano-Gallego L, Cardoso L, Pennisi MG, Petersen C, Bourdeau P, Oliva G, et al. Diagnostic challenges in the Era of canine *Leishmania infantum* vaccines. Trends Parasitol. 2017;33:706–17. 28689776 10.1016/j.pt.2017.06.004

[4] Solano-Gallego L, Miró G, Koutinas A, Cardoso L, Pennisi MG, Ferrer L, et al. LeishVet guidelines for the practical management of canine leishmaniosis. Parasit Vectors. 2011;4:86. 21599936 10.1186/1756-3305-4-86 PMC3125381

[5] Koutinas AF, Polizopoulou ZS, Saridomichelakis MN, Argyriadis D, Fytianou A, Plevraki KG. Clinical considerations on canine visceral leishmaniasis in Greece: a retrospective study of 158 cases (1989–1996). J Am Anim Hosp Assoc. 1999;35:376–83. 10493412 10.5326/15473317-35-5-376

[6] Costa FAL, Goto H, Saldanha LCB, Silva SMMS, Sinhorini IL, Silva TC, et al. Histopathologic patterns of nephropathy in naturally acquired canine visceral leishmaniasis. Vet Pathol. 2003;40:677–84. 14608021 10.1354/vp.40-6-677

[7] Zatelli A, Borgarelli M, Santilli R, Bonfanti U, Nigrisoli E, Zanatta R, et al. Glomerular lesions in dogs infected with *Leishmania* organisms. Am J Vet Res. 2003;64:558–61. 12755294 10.2460/ajvr.2003.64.558

[8] Meléndez-Lazo A, Ordeix L, Planellas M, Pastor J, Solano-Gallego L. Clinicopathological findings in sick dogs naturally infected with *Leishmania infantum*: comparison of five different clinical classification systems. Res Vet Sci. 2018;117:18–27. 29153900 10.1016/j.rvsc.2017.10.011

[9] Cortadellas O, del Palacio MJF, Bayón A, Albert A, Talavera J. Systemic hypertension in dogs with leishmaniasis: prevalence and clinical consequences. J Vet Intern Med. 2006;20:941–7. 16955820 10.1892/0891-6640(2006)20[941:shidwl]2.0.co;2

[10] Koutinas AF, Koutinas CK. Pathologic mechanisms underlying the clinical findings in canine leishmaniosis due to *Leishmania infantum*/*chagasi*. Vet Pathol. 2014;51:527–38. 24510947 10.1177/0300985814521248

[11] Oliva G, Roura X, Crotti A, Maroli M, Castagnaro M, Gradoni L, et al. Guidelines for treatment of leishmaniasis in dogs. J Am Vet Med Assoc. 2010;236:1192–8. 20513197 10.2460/javma.236.11.1192

[12] Roura X, Fondati A, Lubas G, Gradoni L, Maroli M, Oliva G, et al. Prognosis and monitoring of leishmaniasis in dogs: a working group report. Vet J. 2013;198:43–7. 23680263 10.1016/j.tvjl.2013.04.001

[13] Torres M, Bardagí M, Roura X, Zanna G, Ravera I, Ferrer L. Long term follow-up of dogs diagnosed with leishmaniosis (clinical stage II) and treated with meglumine antimoniate and allopurinol. Vet J. 2011;188:346–51. 20594876 10.1016/j.tvjl.2010.05.025

[14] Pierantozzi M, Roura X, Paltrinieri S, Poggi M, Zatelli A. Variation of proteinuria in dogs with leishmaniasis treated with meglumine antimoniate and allopurinol: a retrospective study. J Am Anim Hosp Assoc. 2013;49:231–6. 23690493 10.5326/JAAHA-MS-5840

[15] Cortadellas O, Talavera J, Fernández del Palacio MJ. Evaluation of the effects of a therapeutic renal diet to control proteinuria in proteinuric non-azotemic dogs treated with benazepril. J Vet Intern Med. 2014;28:30–7. 24372810 10.1111/jvim.12246 PMC4895532

[16] Proverbio D, Spada E, de Giorgi GB, Perego R. Proteinuria reduction after treatment with miltefosine and allopurinol in dogs naturally infected with leishmaniasis. Vet World. 2016;9:904. 27651682 10.14202/vetworld.2016.904-908 PMC5021843

[17] Zatelli A, Roura X, D’Ippolito P, Berlanda M, Zini E. The effect of renal diet in association with enalapril or benazepril on proteinuria in dogs with proteinuric chronic kidney disease. Open Vet J. 2016;6:121–7. 27540513 10.4314/ovj.v6i2.8 PMC4980477

[18] Paltrinieri S, Mangiagalli G, Ibba F. Use of urinary γ-glutamyl transferase (GGT) to monitor the pattern of proteinuria in dogs with leishmaniasis treated with *N*-methylglucamine antimoniate. Res Vet Sci. 2018;119:52–5. 29857246 10.1016/j.rvsc.2018.05.014

[19] Pardo-Marín L, Martínez-Subiela S, Pastor J, Tvarijonaviciute A, Garcia-Martinez JD, Segarra S, et al. Evaluation of various biomarkers for kidney monitoring during canine leishmaniosis treatment. BMC Vet Res. 2017;13:31. 28114941 10.1186/s12917-017-0956-0 PMC5259918

[20] Paltrinieri S, Gradoni L, Roura X, Zatelli A, Zini E. Laboratory tests for diagnosing and monitoring canine leishmaniasis. Vet Clin Pathol. 2016;45:552–78. 27805725 10.1111/vcp.12413

[21] Szczepankiewicz B, Pasławska U, Nowak M, Bąchor R, Czyżewska-Buczyńska A, Pasławski R, et al. Early detection of active glomerular lesions in dogs and cats using podocin. J Vet Res. 2019;63:573. 31934669 10.2478/jvetres-2019-0062 PMC6950428

[22] Vogelmann SU, Nelson WJ, Myers BD, Lemley KV. Urinary excretion of viable podocytes in health and renal disease. Am J Physiol Renal Physiol. 2003;285:40–8. 10.1152/ajprenal.00404.2002 PMC3368602 12631553

[23] Szczepankiewicz B, Paslawska U, Paslawski R, Gebarowski T, Zasada W, Michalek M, et al. The urine podocin/creatinine ratio as a novel biomarker of cardiorenal syndrome in dogs due to degenerative mitral valve disease. J Physiol Pharmacol. 2019;70. 10.26402/jpp.2019.2.06 31356184

[24] de Souza C, Coelho M, Antonelo DS, Passarelli D, Rochetti AL, Fukumasu H, et al. Nephrin and podocin mRNA detection in urine sediment of dogs with chronic kidney disease: preliminary observations. J Vet Res. 2022;66:281–8. 35892112 10.2478/jvetres-2022-0019 PMC9281531

[25] Siwińska N, Pasławska U, Bąchor R, Szczepankiewicz B, Żak A, Grocholska P, et al. Evaluation of podocin in urine in horses using qualitative and quantitative methods. PLoS ONE. 2020;15:e0240586. 33057359 10.1371/journal.pone.0240586 PMC7561189

[26] Guo Y, Pace J, Li Z, Ma’ayan A, Wang Z, Revelo MP, et al. Podocyte-specific induction of krüppel-like factor 15 restores differentiation markers and attenuates kidney injury in proteinuric kidney disease. J Am Soc Nephrol. 2018;29:2529–45. 30143559 10.1681/ASN.2018030324 PMC6171275

[27] Wagner N, Wagner KD, Xing Y, Scholz H, Schedl A. The major podocyte protein nephrin is transcriptionally activated by the Wilms’ tumor suppressor WT1. J Am Soc Nephrol. 2004;15:3044–51. 15579507 10.1097/01.ASN.0000146687.99058.25

[28] Lajdova I, Oksa A, Horvathova M, Spustová V. Expression of puninergic P2X7 receptors in subpopulations of peripheral blood mononuclear cells in early-stage of chronic kidney disease. J Physiol Pharmacol. 2017;68:779–85. 29375053

[29] Kasztan M, Jankowski M. Involvement of P2 receptors in regulation of glomerular permeability to albumin by extracellular nucleotides of intra-/extra-glomerular origins. J Physiol Pharmacol. 2016;67:177–83. 27226177

[30] Trimarchi H. Podocyturia: what is in a name? J Transl Int Med. 2015;3:51–6. 27847887 10.1515/jtim-2015-0003 PMC4936448

[31] de Torres MM, Chitarra CS, Nakazato L, de Almeida ABPF, Sousa VRF. Nephrin gene expression in chronic kidney disease of dogs with *Leishmania* (*Leishmania*) *infantum chagasi*. Braz J Infect Dis. 2016;20:516. 27527562 10.1016/j.bjid.2016.07.008 PMC9425462

[32] International Renal Interest Society: IRIS Staging of CKD (modified 2019). International Renal Interest Society, Cambridge, 2019. http://www.iris-kidney.com/pdf/IRIS_Staging_of_CKD_modified_2019.pdf.

[33] Chang JH, Paik SY, Mao L, Eisner W, Flannery PJ, Wang L, et al. Diabetic kidney disease in FVB/NJ Akita mice: temporal pattern of kidney injury and urinary nephrin excretion. PLoS ONE. 2012;7:e33942. 22496773 10.1371/journal.pone.0033942 PMC3319540

[34] O’Brien SP, Smith M, Ling H, Phillips L, Weber W, Lydon J, et al. Glomerulopathy in the KK.Cg-A(y)/J mouse reflects the pathology of diabetic nephropathy. J Diabetes Res. 2013;2013:498925. 23710468 10.1155/2013/498925 PMC3655591

[35] Jim B, Ghanta M, Qipo A, Fan Y, Chuang PY, Cohen HW, et al. Dysregulated nephrin in diabetic nephropathy of type 2 diabetes: a cross-sectional study. PLoS ONE. 2012;7:e36041. 22615747 10.1371/journal.pone.0036041 PMC3355157

[36] Wang Y, Zhao S, Loyd S, Groome LJ. Increased urinary excretion of nephrin, podocalyxin, and βig-h3 in women with preeclampsia. Am J Physiol Renal Physiol. 2012;302:F1084–9. 22301621 10.1152/ajprenal.00597.2011

[37] Jim B, Mehta S, Qipo A, Kim K, Cohen HW, Moore RM, et al. A comparison of podocyturia, albuminuria and nephrinuria in predicting the development of preeclampsia: a prospective study. PLoS ONE. 2014;9:e101445. 25010746 10.1371/journal.pone.0101445 PMC4092019

[38] Bragato N, Borges NC, Fioravanti MCS. B-mode and Doppler ultrasound of chronic kidney disease in dogs and cats. Vet Res Commun. 2017;41:307–15. 28634673 10.1007/s11259-017-9694-9

[39] Perondi F, Lippi I, Marchetti V, Bruno B, Borrelli A, Citi S. How ultrasound can be useful for staging chronic kidney disease in dogs: ultrasound findings in 855 cases. Vet Sci. 2020;7:1–8. 10.3390/vetsci7040147 PMC7712280 33019496

[40] Behr S, Trumel C, Palanché F, Braun JP. Assessment of a pyrogallol red technique for total protein measurement in the cerebrospinal fluid of dogs. J Small Anim Pract. 2003;44:530–3. 14692549 10.1111/j.1748-5827.2003.tb00115.x

[41] Rossi G, Bertazzolo W, Binnella M, Scarpa P, Paltrinieri S. Measurement of proteinuria in dogs: analytic and diagnostic differences using 2 laboratory methods. Vet Clin Pathol. 2016;45:450–8. 27564569 10.1111/vcp.12388

[42] Brown N, Segev G, Francey T, Kass P, Cowgill LD. Glomerular filtration rate, urine production, and fractional clearance of electrolytes in acute kidney injury in dogs and their association with survival. J Vet Intern Med. 2015;29:28. 25594609 10.1111/jvim.12518 PMC4858109

[43] Wang P, Li M, Liu Q, Chen B, Ji Z. Detection of urinary podocytes and nephrin as markers for children with glomerular diseases. Exp Biol Med (Maywood). 2015;240:169–74. 25245074 10.1177/1535370214548995 PMC4935320

[44] Sato S, Yanagihara T, Ghazizadeh M, Ishizaki M, Adachi A, Sasaki Y, et al. Correlation of autophagy type in podocytes with histopathological diagnosis of IgA nephropathy. Pathobiology. 2009;76:221–6. 19816081 10.1159/000228897

[45] Paparcone R, Fiorentino E, Cappiello S, Gizzarelli M, Gradoni L, Oliva G, et al. Sternal aspiration of bone marrow in dogs: a practical approach for canine leishmaniasis diagnosis and monitoring. J Vet Med. 2013;2013:1–4. 10.1155/2013/217314 PMC4590857 26464903

[46] Solano-Gallego L, Rodriguez-Cortes A, Trotta M, Zampieron C, Razia L, Furlanello T, et al. Detection of *Leishmania infantum* DNA by fret-based real-time PCR in urine from dogs with natural clinical leishmaniosis. Vet Parasitol. 2007;147:315–9. 17532143 10.1016/j.vetpar.2007.04.013

[47] Gilbert A, Changjuan A, Guixue C, Jianhua L, Xiaosong Q. Urinary matrix metalloproteinase-9 and nephrin in idiopathic membranous nephropathy: a cross-sectional study. Dis Markers. 2021;2021:1620545. 34707724 10.1155/2021/1620545 PMC8545589

[48] Szczepankiewicz B, Bąchor R, Pasławski R, Siwińska N, Pasławska U, Konieczny A, et al. Evaluation of tryptic podocin peptide in urine sediment using LC–MS-MRM method as a potential biomarker of glomerular injury in dogs with clinical signs of renal and cardiac disorders. Molecules. 2019;24:3088. 31454880 10.3390/molecules24173088 PMC6749423

[49] Ichii O, Yabuki A, Sasaki N, Otsuka S, Ohta H, Yamasaki M, et al. Pathological correlations between podocyte injuries and renal functions in canine and feline chronic kidney diseases. Histol Histopathol. 2011;26:1243–55. 21870328 10.14670/HH-26.1243

[50] Lees GE, Brown SA, Elliott J, Grauer GF, Vaden SL. Assessment and management of proteinuria in dogs and cats: 2004 ACVIM Forum Consensus Statement (small animal). J Vet Intern Med. 2005;19:377–85. 15954557 10.1892/0891-6640(2005)19[377:aamopi]2.0.co;2

[51] Aresu L, Benali S, Ferro S, Vittone V, Gallo E, Brovida C, et al. Light and electron microscopic analysis of consecutive renal biopsy specimens from *Leishmania*-seropositive dogs. Vet Pathol. 2013;50:753–60. 22961886 10.1177/0300985812459336

[52] Suzuki T, Matsusaka T, Nakayama M, Asano T, Watanabe T, Ichikawa I, et al. Genetic podocyte lineage reveals progressive podocytopenia with parietal cell hyperplasia in a murine model of cellular/collapsing focal segmental glomerulosclerosis. Am J Pathol. 2009;174:1675–82. 19359523 10.2353/ajpath.2009.080789 PMC2671256

[53] Hara M, Yanagihara T, Hirayama Y, Ogasawara S, Kurosawa H, Sekine S, et al. Podocyte membrane vesicles in urine originate from tip vesiculation of podocyte microvilli. Hum Pathol. 2010;41:1265–75. 20447677 10.1016/j.humpath.2010.02.004

[54] Nakamura T, Ushiyama C, Suzuki S, Hara M, Shimada N, Ebihara I, et al. The urinary podocyte as a marker for the differential diagnosis of idiopathic focal glomerulosclerosis and minimal-change nephrotic syndrome. Am J Nephrol. 2000;20:175–9. 10878397 10.1159/000013580

[55] Jacob F, Polzin DJ, Osborne CA, Neaton JD, Kirk CA, Allen TA, et al. Evaluation of the association between initial proteinuria and morbidity rate or death in dogs with naturally occurring chronic renal failure. J Am Vet Med Assoc. 2005;226:393–400. 15702689 10.2460/javma.2005.226.393

[56] Lioudaki E, Stylianou KG, Petrakis I, Kokologiannakis G, Passam A, Mikhailidis DP, et al. Increased urinary excretion of podocyte markers in normoalbuminuric patients with diabetes. Nephron. 2015;131:34–42. 26340089 10.1159/000438493

[57] Schwarz K, Simons M, Reiser J, Saleem MA, Faul C, Kriz W, et al. Podocin, a raft-associated component of the glomerular slit diaphragm, interacts with CD2AP and nephrin. J Clin Invest. 2001;108:1621–9. 11733557 10.1172/JCI12849 PMC200981

[58] Huber TB, Köttgen M, Schilling B, Walz G, Benzing T. Interaction with podocin facilitates nephrin signaling. J Biol Chem. 2001;276:41543–6. 11562357 10.1074/jbc.C100452200

[59] Wickman L, Afshinnia F, Wang SQ, Yang Y, Wang F, Chowdhury M, et al. Urine podocyte mRNAs, proteinuria, and progression in human glomerular diseases. J Am Soc Nephrol. 2013;24:2081. 24052633 10.1681/ASN.2013020173 PMC3839551

[60] Martínez-Subiela S, Tecles F, Eckersall PD, Cerón JJ. Serum concentrations of acute phase proteins in dogs with leishmaniasis. Vet Rec. 2002;150:241–4. 11916025 10.1136/vr.150.8.241

[61] Martinez-Subiela S, Cerón JJ, Strauss-Ayali D, Garcia-Martinez JD, Tecles F, Tvarijonaviciute A, et al. Serum ferritin and paraoxonase-1 in canine leishmaniosis. Comp Immunol Microbiol Infect Dis. 2014;37:23–9. 24268430 10.1016/j.cimid.2013.10.004

[62] Silvestrini P, Zoia A, Planellas M, Roura X, Pastor J, Cerón JJ, et al. Iron status and C-reactive protein in canine leishmaniasis. J Small Anim Pract. 2014;55:95–101. 24372300 10.1111/jsap.12172

[63] Braun JP, Lefebvre HP, Watson ADJ. Creatinine in the dog: a review. Vet Clin Pathol. 2003;32:162–79. 14655101 10.1111/j.1939-165x.2003.tb00332.x

[64] Hall JA, Yerramilli M, Obare E, Yerramilli M, Melendez LD, Jewell DE. Relationship between lean body mass and serum renal biomarkers in healthy dogs. J Vet Intern Med. 2015;29:808–14. 25913398 10.1111/jvim.12607 PMC4895404

[65] Kopke MA, Burchell RK, Ruaux CG, Burton SE, Lopez-Villalobos N, Gal A. Variability of symmetric dimethylarginine in apparently healthy dogs. J Vet Intern Med. 2018;32:736–42. 29469955 10.1111/jvim.15050 PMC5867003

[66] Palacio J, Liste F, Gascón M. Enzymuria as an index of renal damage in canine leishmaniasis. Vet Rec. 1997;140:477–80. 9160531 10.1136/vr.140.18.477

[67] Ibba F, Mangiagalli G, Paltrinieri S. Urinary gamma-glutamyl transferase (GGT) as a marker of tubular proteinuria in dogs with canine leishmaniasis, using sodium dodecylsulphate (SDS) electrophoresis as a reference method. Vet J. 2016;210:89–91. 26897435 10.1016/j.tvjl.2016.01.012

[68] Hudson EB, Strombeck DR. Effects of functional nephrectomy on the disappearance rates of canine serum amylase and lipase. Am J Vet Res. 1978;39:1316–21. 697139

[69] Carne T, Scheele G. Amino acid sequences of transport peptides associated with canine exocrine pancreatic proteins. J Biol Chem. 1982;257:4133–40. 6175639

[70] de Schepper J, Capiau E, van Bree H, de Cock I. The diagnostic significance of increased urinary and serum amylase activity in bitches with pyometra. Zentralbl Veterinarmed A. 1989;36:431–7. 2477969 10.1111/j.1439-0442.1989.tb00750.x

[71] Li C, Szeto C-C. Urinary podocyte markers in diabetic kidney disease. Kidney Res Clin Pract. 2024;43:274–86. 38325865 10.23876/j.krcp.23.109 PMC11181047

[72] Zeng L, Szeto CC. Urinary podocyte markers in kidney diseases. Clin Chim Acta. 2021;523:315–24. 34666027 10.1016/j.cca.2021.10.017

[73] Mesfine BB, Vojisavljevic D, Kapoor R, Watson D, Kandasamy Y, Rudd D. Urinary nephrin—a potential marker of early glomerular injury: a systematic review and meta-analysis. J Nephrol. 2023;37:39–51. 36808610 10.1007/s40620-023-01585-0 PMC10920435

